# Extrusion-Based 3D Printing of Ceramic Pastes: Mathematical Modeling and In Situ Shaping Retention Approach

**DOI:** 10.3390/ma14051137

**Published:** 2021-02-28

**Authors:** Fuwen Hu, Tadeusz Mikolajczyk, Danil Yurievich Pimenov, Munish Kumar Gupta

**Affiliations:** 1 School of Mechanical and Material Engineering, North China University of Technology, Shijingshan Jinyuanzhuang Road 5, Beijing 100144, China; 2 Department of Production Engineering, UTP University of Science and Technology, Al. prof. S. Kaliskiego 7, 85-796 Bydgoszcz, Poland; tami@utp.edu.pl; 3 Department of Automated Mechanical Engineering, South Ural State University, Lenin Prosp. 76, 454080 Chelyabinsk, Russia; danil_u@rambler.ru (D.Y.P.); munishguptanit@gmail.com (M.K.G.); 4 Key Laboratory of High Efficiency and Clean Mechanical Manufacture, Ministry of Education, School of Mechanical Engineering, Shandong University, Jingshi Road 17923, Jinan 250061, China

**Keywords:** ceramic, extrusion, additive manufacturing, shaping capacity, printability, drying

## Abstract

Extrusion-based three-dimensional (3D) printing methods are preferred and emerging approaches for freely digital fabrication of ceramics due to ease of use, low investment, high utilization of materials, and good adaptability to multi-materials. However, systematic knowledge still lacks an explanation for what is their 3D printability. Moreover, some uncontrollable factors including extrudate shape retention and nonuniform drying inevitably limit their industrial applications. The purpose of this research was to present a new shaping retention method based on mathematical synthesis modeling for extrusion-based 3D-printing of ceramic pastes. Firstly, the steady-state equilibrium equation of the extrusion process was derived to provide clearer theoretical indications than purely experimental methods. Furthermore, a mathematical description framework was synthesized to better understand the extrusion-based 3D-printing of ceramic pastes from several realms: pastes rheology, extrudability, shape-holdability, and drying kinetics. Secondly, for eliminating shaping drawbacks (e.g., deformation and cracks) originating from non-digital control factors, we put forward a digital shape-retention technology inspired by the generalized drying kinetics of porous materials, which was different from existing retention solutions, e.g., freezing retention, thermally induced gelation, and using removable support structures. In addition, we developed an in situ hot air flow drying device easily attached to the nozzle of existing 3D printers. Confirmatory 3D-printing experiments of thin-walled cone-shape benchmark parts and the fire arrowhead-like object clearly demonstrated that the presented shape-retention method not only upgraded layer-by-layer forming capability but also enabled digital control of extrudate solidification. In addition, many more experimental results statistically showed that both fully solid parts and purely thin-wall parts had higher dimensional accuracy and better surface quality than the offline drying method. The 3D printed ceramic products with complex profiled surfaces conceivably demonstrated that our improved extrusion-based 3D-printing process of ceramic pastes has game-changing potentials beyond the traditional craftsmanship capacity.

## 1. Introduction

Over more than 30 years, three-dimensional (3D) printing or additive manufacturing (AM) has evolved beyond rapid prototyping and, more recently, has demonstrated great potential for mass customization production [1]. Since 3D printing is changing how and for who products are manufactured as well as when and where they are produced, additive manufacturing has been viewed as a landmark event of the new industrial revolution [2]. With reference to the definition of AM from ISO/ASTM 17296 standard [3], AM is a variety of near net shaping processes of layer-by-layer building components driven by three-dimensional model data, absolutely different from traditionally subtractive or formative manufacturing methodologies [4]. According to the standard, AM processes can be divided into seven categories: material extrusion (ME), material jetting, direct energy deposition (DED), binder jetting, sheet lamination, powder bed fusion, and vat photopolymerization [5]. Technically speaking, currently existing AM techniques have been employed for the shaping of ceramic precursors or green ceramic objects [6], except that the Selective Laser Melting (SLM) method [7] and DED method [8] have successfully printed ceramic components. In the comprehensive review survey written by Travitzky et al., a more instinctive categorization of commercialized AM processes used for ceramic components was presented in the light of dimensional order, commercial accessibility, state of aggregation of their starting material, and layer formation principles. It should be mentioned that some research suggested that fully ceramic net-shaped specimens of almost 100% densities can be produced via SLM or DED [9], but thermal shocks, melting-induced pores, and other challenges still remained due to the unstable and nonlinear laser–powder interaction [10]. Therefore, each method for additively shaping ceramics has its own superiority and restrictions. The key problem seems to rely on the coupling of a specific AM technology with the formulation of corresponding feedstocks, which would especially undertake the fabrication of dense ceramic components with desirable properties (density, mechanical strength, surface finish, etc.).

This research mainly involved the extrusion-based 3D-printing methodology of ceramic components. Generally, the ME process refers to additively forming processes in which the material is layer-by-layer dispensed through a nozzle onto the platform until the 3D object is formed. However, this standard definition of ME only highlights the feedstock dispenser and does not reflect the material solidification methodologies. Therefore, many types of ME processes that have been developed may easily lead to confusion, such as Aqueous-Based Extrusion Fabrication (ABEF) [11], Fused Deposition of Ceramics (FDC) [12], Robocasting (RC) [13], Freeze-form Extrusion Fabrication (FEF) [14], Direct Ink Writing (DIW) [15], and Ceramic On-Demand Extrusion (CODE) [16]. An important distinction between all these technological synonyms is the mechanism of feedstock solidification. In addition, ME in the context of 3D printing is very different from the traditional extrusion process that used to produce parts in bulk materials with constant cross sections [17]. The traditional extrusion process may facilitate the former in some technical aspects, but it is far from a digitally layer-by-layer extrusion forming technology and it also does not need to deal with the issue of shape retention.

To our best knowledge, systematic knowledge still lacks an explanation for what the full 3D printability of the extrusion-based 3D-printing of ceramics is. For instance, most documents [16,18,19] focusing on basic process parameters setup are experimental and fragmented. Especially when it comes to the ability to make monolithic parts, size precision, and surface quality, it still requires a lot of theoretical analysis and experimental research. The motivation of this work was to build a synthetically mathematical modeling and to present an in situ shaping retention method for the extrusion-based 3D-printing of ceramic pastes.

In the next section, we first review the research status of extrusion-based 3D-printing processes, with this review instructing us on how to seek a trade-off between the theoretical complexity and technical promotion of extrusion. In the third section, a geometrically mathematical model for extrusion process of ceramic paste was built using the infinitesimal method, and from this analytical formulation, we attempted to provide a theoretical guidance for extrusion behavior control. In the fourth section, to address shaping flaws, e.g., collapse during deposition and cracks during drying, we mainly studied the in situ hot air-drying method from device design to experimental verification. Furthermore, we studied the sinterability and sintering shrinkage of 3D-printed thin-wall green bodies.

## 2. Theoretical Complexity and Enabling Schemes of Extrusion-Based 3D-Printing

In this section, we review related research work of extrusion-based 3D-printing processes from two contexts: the complexity of theoretical perspective and the enabling schemes of technical perspective.

### 2.1. Complexity of Theoretical Modeling for Extrusion-Based 3D-Printing Process

Extrusion-based 3D printing has been widely applied for many different material-forming processes including food, ceramic, chemical, and pharmaceutical materials, where pastes with high-volume fraction of solids exhibit a complex rheological behavior. Undoubtedly, the paste material properties and paste flow are key points for shaping quality in the extrusion-based layered shaping process, which influences shape retention and extrudability. In recent years, many researchers and scientists coined the term “3D printability” [18,20] or “printability” [21] to describe this complex nonlinear relationships among the pastes rheology, extrudability, shape-holdability during deposition, and processing parameters. In other words, theoretical modeling for extrusion-based layered shaping process is confronted with unprecedented complexity including the still inadequate description of pastes rheology and drying kinetics [22].

The most commonly rheological framework for ceramic pastes is the six-parameters Benbow–Bridgwater model consisting of a static interrelation between extrusion velocity and extrusion force, i.e., they are formulated by an algebraic equation rather than a differential equation [10]. The model beneficially balances the contradiction between complexity and accuracy. When the paste extrusion is in steady state, this model maybe effectively estimates the relationship between extrusion pressure and extrusion technological parameters [22]. For example, the research of Guilherme et al. [23] illustrated a good agreement between the measured values and those predicted by the Benbow–Bridgwater model. However, when a force was exerted to the paste to move it through the die of an extruder, the extrusion stress is split between the friction at the inter particle contacts and the viscous shear in the liquid binder [24]. The role of the liquid phase is to surround and separate the particles from each other and to promote the particles to slide against each other. During extrusion, the relative movement between liquid phase and solid particles would cause “liquid phase migration (LPM)”, the variations in liquid content in pastes. Liquid phase migration changes the material composition, causing the ratio of solids to liquid volume to increase. The rheology of the pastes highly relies upon the local liquid volume fraction, and LPM therefore changes the flow regime in the extruder. Moreover, two major disturbances are very likely to take place: agglomerate breakdown and air bubble release. Their size and distribution are both random. The sudden increase in extrusion force caused by the breakdown of agglomerates and the extrusion force drop led by air bubble release cannot be dependably predicted. These above mentioned factors and the non-Newtonian behavior, compressibility, and inhomogeneity of ceramic pastes strongly reveal the complexity of theoretical modeling for the extrusion process dynamics.

Stuecker et al. [13] investigated the rheology control method for robocasting near-net shape parts without significantly altering solid-loading suspension. The research work of Tang et al. [19] presented that the influential degree of processing parameters on the dimensional deviation of formation was in the following order: solid loading, layer height, print speed, and nozzle diameter. The simulation work of Patel et al. [24] revealed that there was a complex relationship among extrusion geometry, extrusion speed, and LPM. Bardesley and coworker [25] provided a methodology to evaluate liquid phase migration. Liu et al. [26] studied the effect of liquid phase migration on the extrusion of aqueous alumina pastes, and their research suggested the occurrence of liquid phase migration was strongly dependent on the ram velocity.

### 2.2. Technical Solutions for Extrusion-Based 3D-Printing Process

From a technical perspective, there are two aspects for enabling technology solutions for a variety of extrusion-based layered shaping processes: the feedstock extrusion mechanism and the shape-retention method.

Roughly speaking, the extrusion mechanisms can be divided into syringe-based extrusion, screw-based extrusion, and pneumatic extrusion. The experimental research of Li et al. [16] indicated that the needle valve and screw valve-based methods are more reliable at the start and stop of extrusion than the ram extruder-based method, while the screw valve-based method is more stable in continuous production than the ram extruder and needle valve-based method. The comparative study by Guo et al. [27] between syringe-based and screw-based extrusion 3D printing suggested that the screw-based method was inappropriate for printing inks with high viscosity. Masuda et al. [28] remodeled the popular Fused Deposition Modeling (FDM) type extruder using a syringe to fabricate large SiC ceramics with complex shapes. In the Ceramic On-Demand Extrusion (CODE) process developed by Ghazanfari et al. [29], they used an auger valve as the extrusion subsystem. In the research of Mason et al. [11], their extrusion device was a plunger driven by a linear actuator. In robocasting process [13], the creators also used a syringe-based extruder.

Technically, the second enabling technology is the shape-retention method. Shape retention directly affects the size, shape, surface quality, and efficiency of the extrusion-based layered shaping. Undoubtedly, the most common shape-retaining method is natural evaporation of the solvent to induce dilatancy of the suspension to maintain the shape [11,13]. Besides natural drying, researchers explored many other shape-retention methods and claimed that they developed new extrusion-based layered shaping processes different from each other. For instance, the extrudate in the Freeze-form extrusion fabrication (FEF) was deposited on a platform or previously formed layer in a freezer space and was rapidly frozen solid. During the Ceramic On-Demand Extrusion (CODE) process, the pastes were extruded at room temperature and each layer was solidified via partial drying using an infrared lamp, with a liquid oil surrounding the part. Biswas et al. [30] presented the shape-retention concept of thermally induced gelation of methyl cellulose; that is to say, the parts were simultaneously exposed to hot air during printing, causing immediate gelation of methyl cellulose undertaking retention of shapes. Ren et al. [31] claimed that they provided a 3D gel-printing (3DGP) process with the bridge between the low cost of the Direct Inkjet Printing (DIP) process and the fast solidification of gelation. Scheithauer et al. [32] put forward the thermoplastic 3D-printing process with a combination of FDM and robocasting, which utilized phase changing techniques to maintain the extrudate shape. In addition to the above mentioned single-extrudate retention methods, the approaches of using water dissolvable materials [33] or easily removed materials as support structures [34] also have been explored for fabricating more complex and precise structures.

## 3. Mathematical Representations of Extrusion-Based 3D-Printing Process

**Figure 1:**
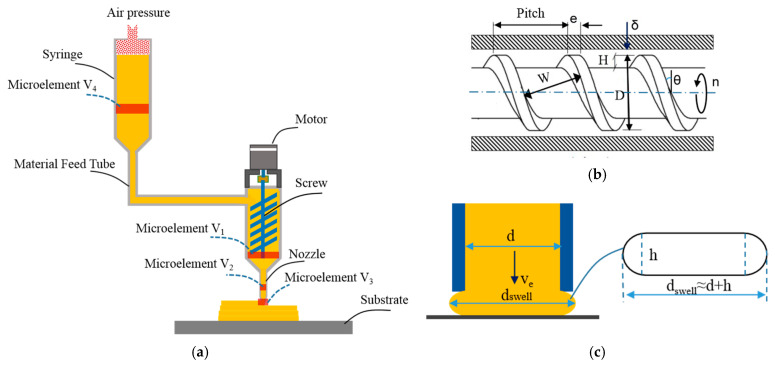
Schematic of extrusion-based three-dimensional printing of ceramic paste: (**a**) static differential model of the extrusion process, (**b**) geometric parameters of auger, and (**c**) the desirable shape of an extrudate.

As mentioned above, the extrusion process of high solid loading ceramic pastes is extremely complex and dynamic. For all this, building the mathematical model of this process is still the first step to clearly understanding the influences of layer height, paste extrusion speed, scanning speed, and other process parameters on the deposition forming state. Here, we assume that the ceramic pastes are homogenous and incompressible and that the extrusion processes have smooth and steady-state material flow. That is to say, the elastic deform of viscoelastic paste, viscosity-dependent volumetric flow rate, and the friction between the paste and the extruder wall are also ignored. Based on the above assumptions, in the unit time during the extrusion process, the flow volumes in the extruder, nozzle, extruded tip, as well as feedstock barrels are equivalent. As illustrated in Figure 1a, they are respectively denoted with $V_{i}$, with i = 1, 2, 3, and 4.

Firstly, according to the drag flow formula of a single screw [35], the microelement $V_{1}$ in the extruder can be expressed as follows:

(1) $$V_{1}=Q_{d}t=\frac{\pi }{2}\mathrm{WHDn}\cos\theta t$$

where n is the screw rotating speed, θ is the screw helix angle, D is the diameter of screw, W is the channel width of screw, H is the flight depth, and n is the rotation speed of a screw; see Figure 1b.

Secondly, we use $v_{e}$ to represent the paste extrusion speed and use d to represent the diameter of nozzle. Then, the microelement $V_{2}$ in the nozzle can be calculated by the following:

(2) $$V_{2}=\frac{v_{e}{t\pi d}^{2}}{4}$$

See Figure 1c. According to an approximation of the extrudate cross section, the microelement $V_{3}$ in extruded tip can be approximated as follows:

(3) $$V_{3}=v_{\mathrm{xy}}\left(\mathrm{hd}+\frac{{\pi h}^{2}}{4}\right)t$$

where $v_{\mathrm{xy}}$ is the 3D printing scanning velocity.

Next, from the equivalent relation between $V_{1}$ and $V_{2}$, the relationship between paste extrusion speed $v_{e}$ and the screw rotating speed n can be easily deduced as follows:

(4) $$v_{e}=\frac{2\mathrm{WHD}\cos\theta }{d^{2}}n$$

Similarly, from the equivalent relation between $V_{2}$ and $V_{3}$, it can be proved out that the filament extrusion velocity $v_{e}$ and the scanning velocity $v_{\mathrm{xy}}$ are compliant with the following:

(5) $$\frac{v_{\mathrm{xy}}}{v_{e}}=\frac{{\pi d}^{2}}{4\mathrm{hd}+{\pi h}^{2}}=\frac{1}{\frac{4}{\pi }\cdot \frac{h}{d}+{\left(\frac{h}{d}\right)}^{2}}$$

In order to more conveniently identify the relationships between layer height h, paste extrusion speed $v_{e}$, and scanning speed $v_{\mathrm{xy}}$, Equation (5) can be further reformulated as follows:

(6) $${\left(\frac{h}{d}\right)}^{2}+\frac{4}{\pi }\cdot \frac{h}{d}-\frac{v_{e}}{v_{\mathrm{xy}}}=0$$

Clearly, Equation (6) is a quadratic equation about h/d; use the root solution method and the following equation can be calculated:

(7) $$h=\frac{\sqrt{4+\pi^{2}\cdot \frac{v_{e}}{v_{\mathrm{xy}}}}-2}{\pi }\cdot d$$

**Figure 2:**
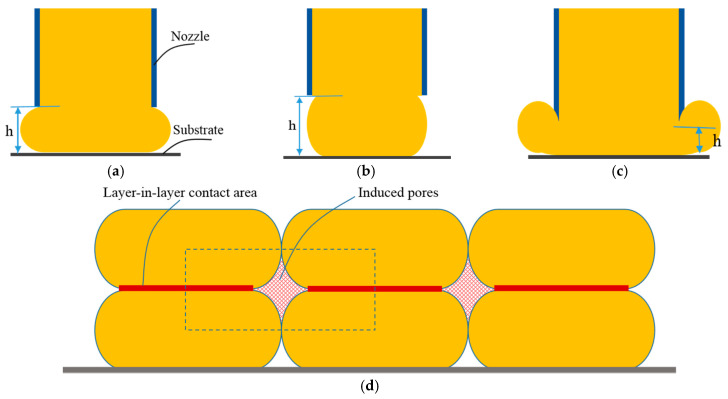
Geometries of the extrudate shapes and layer-in-layer adhesion: (**a**) If the layer height is suitable, then it has a high layer-in-layer contact and a small layer-in-layer gap. (**b**) If the layer height is too big, then it has a low layer-in-layer contact and big layer-in-layer gap. (**c**) If the layer height is too small, then it has an irregular extrudate shape and big size error. (**d**) The ideal layer-in-layer contact area and the induced pores.

Here, we consider Equation (7) as the flowrate equilibrium equation of the extrusion process. This derived equilibrium equation theoretically revealed the restrictive relations of the basic parameters h, $v_{e}$ and $v_{\mathrm{xy}}$. It provides clearer insights than the purely experimental methods [16,19]. Geometrically, these three process parameters, h, $v_{e}$ and $v_{\mathrm{xy}}$, directly affect the extrudate shapes and layer-in-layer adhesion, as shown in Figure 2.

From Figure 2, if given h ≈ d, we can deduce that the filament extrusion velocity $v_{e}$ and the scanning velocity $v_{\mathrm{xy}}$ should be compliant with the following:

(8) $$\frac{\pi +1}{\pi }=\frac{v_{e}}{v_{\mathrm{xy}}}$$

In this case, the gap in induced pores reaches maximum; in contrast, the layer-in-layer contact area is at minimum. Obviously, this leads to the worst conditions for density, porosity, sintering shrinkage, and strength of the formed part. From Figure 2b, we can see that, if the layer height is too big, a low layer-in-layer contact results. This leads to layer-in-layer landslide and even uncontrollable collapse. Therefore, the maximum limit value of the layer height is recommended to be less than 0.9d according to our experimental work.

Otherwise, if given h≪d, theoretically, from Equation (7), $v_{e}$≪$v_{\mathrm{xy}}$ should be satisfied. However, in order to provide enough power to extrude the feedstocks, $v_{e}$ has a minimum limit, here denoted by $v_{e-\min}$. Meanwhile, v_xy_ has a maximum limit value, here denoted by $v_{\mathrm{xy}-\max}$. Therefore, $v_{e}$≪$v_{\mathrm{xy}}$ should not be established. As illustrated in Figure 2c, this leads to irregular and uncontrollable extrudate shapes and the size error becomes very large. In other words, theoretically, the layer height has a minimum limit, which can be defined as follows:

(9) $$h_{\min}=\frac{\sqrt{4+\pi^{2}\times \frac{v_{e-\min}}{v_{\mathrm{xy}-\max}}}-2}{\pi }\times d$$

Concluding, the layer height h should be defined in [$h_{\min}$,0.9d]. In this case, we also can calculate the theoretical porosity of induced pores by the following:

(10) $$V_{\mathrm{porosity}}=\frac{A_{\mathrm{gap}}}{A_{\mathrm{normal}}}=\frac{\left(1-0.25\pi \right)h}{d+h}$$

Clearly, Equation (7) contributes to roughly evaluating the influences of the defined value of layer height on the physical properties of shaped parts. Therefore, suitable definitions of layer height, paste extrusion speed, and scanning speed can produce desirable extrudate shapes and high-quality layer-in-layer adhesion. Otherwise, irregular and even noncontinuous extrudate shapes occur. Using the above mathematical modeling and analysis and further through printing experiments verification, the actual optimal process parameters of a specific extrusion-based 3D-printing system of certain pastes could be proportionally obtained.

There is no doubt that the above mathematical analysis assumes that the feedstocks have good extrudability. In order to control the extrusion process accurately and stably, the influence of slurry properties on the flow behaviors and the extrusion process should be effectively estimated.

First, we introduce the Hagen–Poiseuille Equation [36]:

(11) $$Q_{d}=\frac{{\pi d}^{4}\Delta P}{128{\mu L}_{\mathrm{Nozzle}}}\;\mathrm{or}\;\mu =\frac{{\pi d}^{4}\Delta P}{128Q_{d}L_{\mathrm{Nozzle}}}$$

where $L_{\mathrm{Nozzle}}$ is the length of the nozzle, μ is the viscosity of the pastes, and ΔP is the pressure difference between the end and the top of the nozzle. ΔP can calculated by the following:

(12) $$\Delta P=P_{\mathrm{Screw}}-P_{\mathrm{atm}}=\frac{6{\pi \mu L}_{\mathrm{Screw}}\mathrm{Dn}}{H^{3}\tan\theta }-P_{\mathrm{atm}}$$

where $P_{\mathrm{Screw}}$ is the pressure at the end head of screw, $P_{\mathrm{atm}}$ is the standard atmospheric pressure, and $L_{\mathrm{Screw}}$ is the length of the screw. Obviously, by considering Equations (11) and (12), it is easy to build the relationship between the slurry viscosity μ and the key control parameter, the rotation speed of screw n.

In the extrusion process, the paste flow can be treated as the viscous-plastic body. According to the recommendation [19], in ideal steady flow status, the shearing strain rate $\dot{\gamma }$ can be calculated by the following:

(13) $$\dot{\gamma }=\frac{32Q_{d}}{{\pi d}^{3}}$$

The dependence of shear stress τ on shear strain rate $\dot{\gamma }$ can be further expressed by the simplified constitutive equation [22]:

(14) $$\tau =\tau_{b}+{\mu \dot{\gamma }}^{m}$$

where $\tau_{b}$ is the yield stress of paste. The power exponent m is less than 1.0 for the shear thinning property of pastes. Equations (7), (11) and (12) mathematically reveal that the extrudability is a computable adaptability of different solid loading pastes with a screw-based extrusion mechanism.

As shown in Figure 1c, once the pastes are extruded out of the tip of nozzle, the final diameter of the viscous-plastic filament becomes a little bigger than the nozzle diameter due to loss of the wall shear stress. The swelled extrudate diameter can be calculated by the Phan–Thien–Tanner model [37]:

(15) $$d_{\mathrm{swell}}=\sqrt[{6}]{1+\frac{\tau_{\mathrm{wall}}^{2}}{2G_{0}^{2}}}\times d$$

where $\tau_{\mathrm{wall}}$ is the wall shear stress and $G_{0}=\eta_{0}/\lambda$, where λ is the relaxation time and $\eta_{0}$ is the zero-shear viscosity. Thus, the extrudate swell is mainly affected by the filament extrusion velocity $v_{e}$ and the nozzle configuration. The nozzle with a short length may cause a large amount of swell. The wall shear stress depends on the geometry and the pressure gradient, and it can be calculated by the following [38]:

(16) $$\tau_{\mathrm{wall}}=\frac{\Delta \mathrm{Pd}}{4L_{\mathrm{Nozzle}}}$$

## 4. Limitations of Extrusion-Based 3D-Printing without Shape-Holding Strategies

In the previous section, we derived the equilibrium equation of an extrusion process and explained the suitable definitions of process parameters. However, the real extrusion layered forming process is also affected by other factors such as inevitably inhomogeneity, layered gravity increments, liquid phase migration, and surface tensions. These factors have an especially strong effect on shape retention during layered accumulation. Next, we carried out experimental work based on our self-developed extrusion-based 3D printer [39,40] of ceramic pastes to investigate these limitations.

### 4.1. Experimental Conditions and Data Preparation

For intuitively assessing the shape-retention ability of an extrusion-based 3D-printing system, the conical thin-walled model was selected as benchmark parts, as shown in Table 1. When printing this type of benchmark, the influences of infill density, air gap, and other factors can be excluded; therefore, it is suitable to test the shape-retention ability. The extruder nozzle diameter is 1.0 mm, and its length $L_{\mathrm{Nozzle}}$ is 25 mm. The scanning speed $v_{\mathrm{xy}}$ is 15 mm/s.

The solid loading of kaolin-based pastes available for our extruding device could range between 40 vol.% and 50 vol.%. Our experimental pastes were the kaolin-based pastes with 49 vol.% solids load of mineralogical compositions: quartz 5 wt.%, kaolinite 76 wt.%, illite 8 wt.%, and microcline 11 wt.%. The medium value of the particle size distribution (D50) is 4.8 µm. Uniform and stable pastes were obtained by mixing the mixture for 12 h. The zero-shear viscosity is approximated as 180 Pa·s within the relaxation time 0.1 s, and the yield stress of pastes is 0.035 MPa close to the similar pastes [25]. The effect of paste pH was not considered in this study. As shown in Table 1, the theoretical prediction parameters were estimated by Equations (1)–(16) in steady extruding state. Therefore, the driving parameters should be advisably regulated based on estimates.

### 4.2. Weakness of Shape-Retention Ability

**Figure 3:**
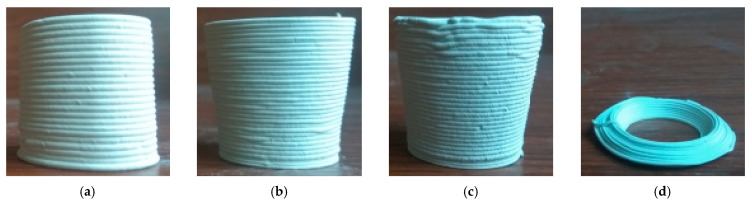
Conical thin-walled benchmark parts with layer height as 0.8d: (**a**) cone angle 0°, (**b**) cone angle 10°, (**c**) cone angle 15°, and (**d**) cone angle 20°.

First, we set up the layer height as 0.8d and printed the conical thin-walled benchmark parts as shown in Figure 3. Clearly, it could be seen that, when the cone angle was up to 15°, the part surface was no longer smooth and tended to collapse. When the cone angle was up to 20°, the part collapsed so quickly that it could not be finalized.

**Figure 4:**
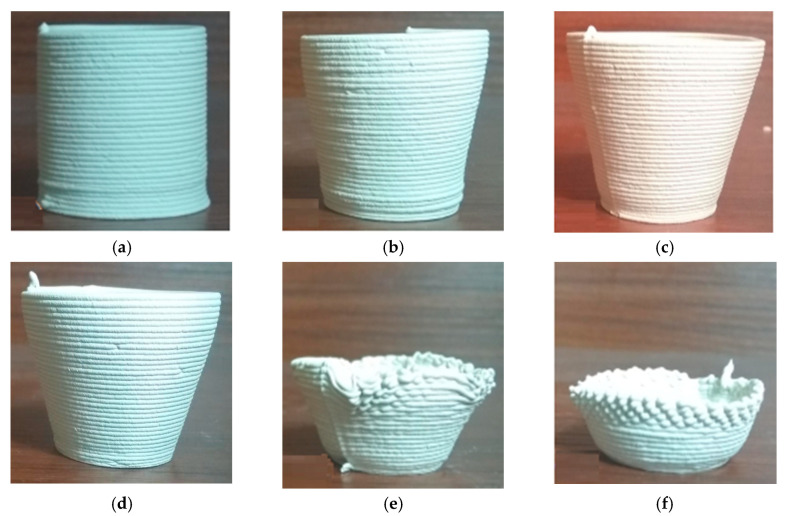
Conical thin-walled benchmark parts with layer height as 0.7d: (**a**) cone angle 0°, (**b**) cone angle 10°, (**c**) cone angle 15°, (**d**) cone angle 20°, (**e**) cone angle 25°, and (**f**) cone angle 30°.

Furthermore, we defined the layer height as 0.7d and printed the conical thin-walled model benchmark parts with the cone angles as 0°, 10°, 15°, 20°, 25°, and 30°. As suggested in Figure 4, when the cone angle was up to 25°, the part collapsed so badly that it could not be finalized.

Obviously, to reduce the layer height, we can extend the forming capacity to certain extent. However, further reducing the layer height also causes an increase in the printing period. The effect of process parameter optimization is very limited for improving the shape-retention ability.

**Figure 5:**
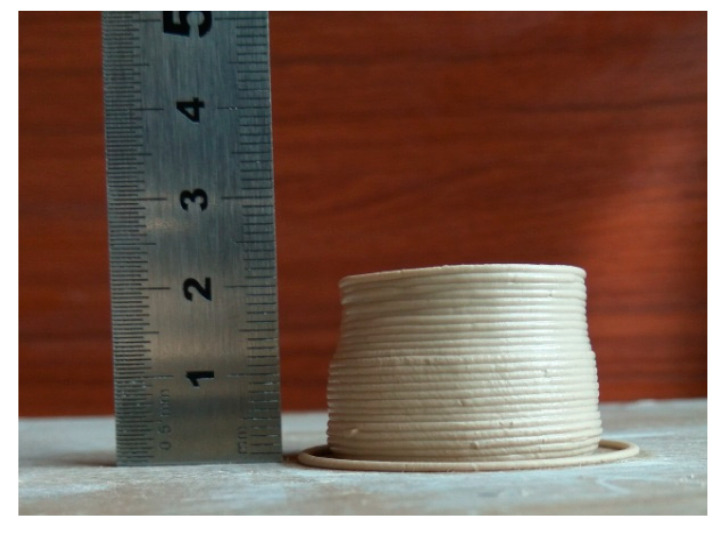
A solid cylinder part with the diameter 30mm and height 20 mm.

In another experiment, we printed a fully solid cylinder part with a diameter 30 mm and height 20 mm. From Figure 5, we could observe that the surface of the workpiece underwent severe deformation.

Comprehensively speaking, ceramic paste is a non-Newtonian fluid with a large water content [10,16,22]. During layer-by-layer forming, the ceramic paste cannot be solidified immediately. Especially under the combined effects of layered gravity increments and liquid phase migration, the layer-in-layer adhesion and the strength of the previous layer cannot support the currently deposited layer, which would inevitably lead to the accumulation of errors until collapses or deformations happen.

### 4.3. Crack Formation Due to Nonuniform Drying

**Figure 6:**
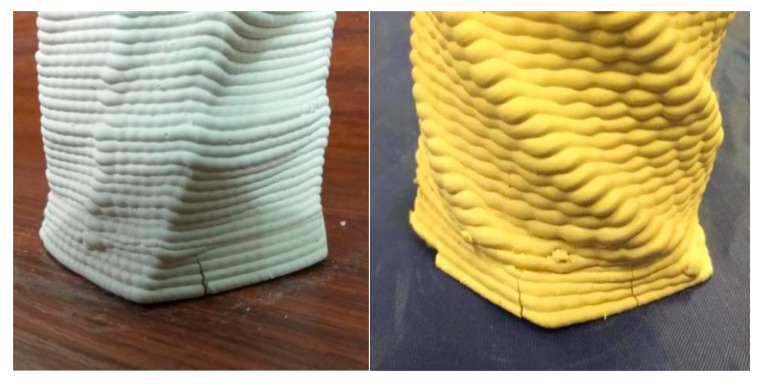
Cracks due to nonuniform drying.

After extrusion, most (more than 90%) of the liquid phase need to be removed to avoid damage by successive drying. However, the drying behavior is a very complex process that involves simultaneous heat and mass transfer to and from the moist porous material. The most important point is that mechanical stresses occur within the material during drying due to water removal and shrinkage. This may cause warping or cracks in the final product if the drying conditions are not carefully chosen and controlled [41]. In our initial experiments with naturally drying to remove 98 wt.% water content for more than 48 h, during this long and uncontrollable process, cracks often occurred as suggested in Figure 6.

Evidently, during this extrusion-based layered shaping process, the shape is maintained and the fully part drying are beyond the digital control of material deposition, thus becoming a technical problem that limits the system’s shaping capacity and forming quality. Therefore, next, we describe the drying kinetics of porous materials and then put forward an online controllable drying solution for the above shaping drawbacks.

## 5. Extrusion-Based 3D-Printing Method with In Situ Hot Air Flow Drying

### 5.1. Drying Kinetics of Porous Materials

Traditionally, fully convective drying of ceramic green bodies is an essential process step in their manufacture. Understanding the drying mechanisms first starts with the movements of liquid within porous ceramics [41]. In an environment with constant conditions, three different stages corresponding to different drying rates are divided. At the first stage, called the constant-rate period, evaporation occurs at the liquid/air interface. During the second stage, named the first falling-rate period, evaporation still occurs from the surface but no longer behaves as a free water surface. The final step is the second falling-rate period, with a nonlinear relation between drying rate and moisture content. During this final period, evaporation occurs within the porous body so that the vapor reaches the surface by diffusing through the pores.

Along with the decrease in the moisture content, the solid content, dimensions, shape, and rheology of the depositing filament change simultaneously. In early drying, the depositing slurry is fluid enough that any constrained stress is immediately relieved by viscous flow [42]. As drying proceeds, the differential shrinkage between the top and bottom of the layer due to the moisture gradient across the thickness of the drying layer occurs. Another driving force is the capillary tension in the pore liquid, which varies in the thickness direction if the evaporation rate is fast relative to the transport rate of the liquid [43]. This pressure gradient may cause warping or cracking if the part body is not stiff or strong enough. Additionally, the complex shapes and structures of depositing parts including protrusions, edges and corners, and wall thickness also affect the moisture gradient redistribution. When the stresses caused by nonuniform shrinkages exceed the fracture resistance of the extrudate, visible or hidden cracks occur.

Obviously, the total stress values dynamically change during drying that depends on layer thickness, surface tension, evaporation rate, viscosity, permeability, solids loading, etc. To avoid cracks, the evaporation rate and drying time should be adjusted according to the given layer thickness and paste properties [29]. According to Chiu’s research [43], there exists a critical cracking thickness $h_{c}$ above which a biaxially stressed layer will crack even without moisture gradient. $h_{c}$ is a function of the magnitude of the biaxial stress $\sigma_{\mathrm{cap}}$ and the fracture resistance ($G_{c}$) of the layer material:

(17) $$h_{c}={\left(\frac{G_{c}}{1.4\sigma_{\mathrm{cap}}}\right)}^{2}$$

Therefore, the critical thickness will only depend on the magnitude of the capillary stress and the fracture resistance. The quantitative analysis of $h_{c}$ and $G_{c}$ is far beyond the scope of this work, but the focus research of granular ceramic films [44] can provide an approximate basis for our demonstration. Most other changes in extrudate properties or drying conditions affect both the capillary pressure and the fracture resistance. The capillary pressure at 100% saturation can be approximated by the following [42]:

(18) $$\sigma_{\mathrm{cap}}=\frac{2\gamma_{\mathrm{lv}}\cos\theta }{r_{\mathrm{pore}}}$$

(19) $$r_{\mathrm{pore}}\approx \frac{2\left(1-\varphi \right)}{\varphi \rho S}$$

where φ, ρ and S are, respectively, the volume fraction, specific surface area, and theoretical density of solid particles; $\gamma_{\mathrm{lv}}$ is the surface tension of water, $r_{\mathrm{pore}}$ is the pore radius and can be approximated using hydraulic radius; and θ is the water contact angle on solid particles.

### 5.2. In Situ Hot Air Flow Drying Device

**Figure 7:**
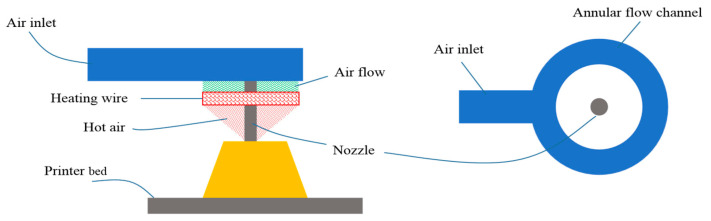
Schematic of in situ hot air flow drying.

As presented in Figure 7, the in situ hot air flow drying device mainly consists of three parts: fan, annular flow channel, and heating wire. The fan provides flowing air, and the heating wire heats the air flow. The heated airflow is blown towards the material deposition spot. Since this device is attached with a nozzle, the extrudate can reduce water content as quickly as possible. This first diminishes the moisture gradient distribution to avoid nonuniform shrinkage. Furthermore, according to Equations (17) and (18), this will decrease the surface tension of water $\gamma_{lv}$, thereby heightening the critical cracking thickness $h_{c}$. Collectively the online hot air drying will expectedly eliminate the cracks.

**Figure 8:**
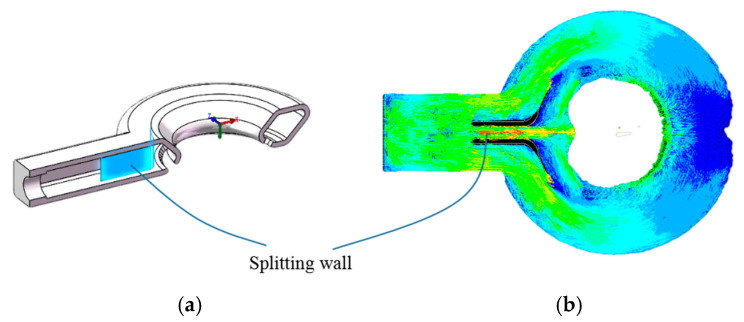
Structural design of annular flow channel: (**a**) sectional view and (**b**) airflow hydrodynamics simulation.

The key to the whole device is to enable the air to blow out uniformly from the annular flow channel to achieve the purpose of uniformly heating the ceramic paste. Therefore, in the structural design of annular flow channel, we used the finite element simulation method of fluid mechanics to optimize and verify the structure. As shown in Figure 8, after design a splitting wall in the inlet, air flow blows out more uniformly along the annular flow channel.

**Figure 9:**
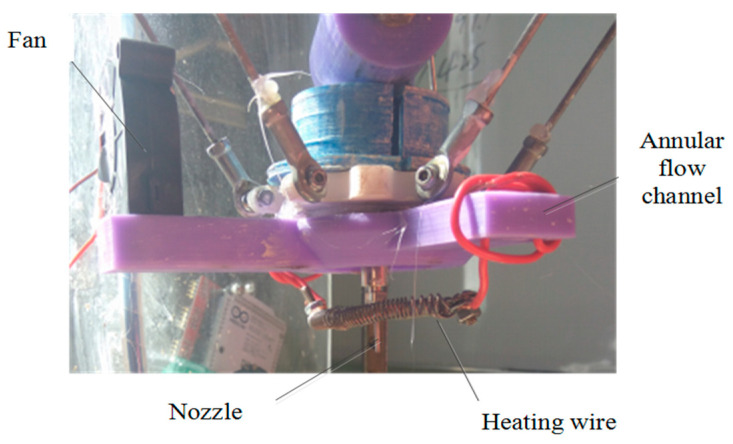
Device for in situ hot air flow drying.

Figure 9 demonstrated the actual in situ hot air flow drying device. Different from current shape-retention solutions, e.g., freezing retention, thermally induced gelation, and radiation drying, this presented solution has many advantages as follows:

- The in situ hot air flow drying device is easy to install on the existing 3D printer without any big revamp.
- The heating capacity of this in situ hot air flow drying device can be numerically controlled by programming the speed of fan and the temperature of heating wire. Therefore, this device can partly adapt to different solids loading pastes or different shaping processes [40].
- The main components of this device, i.e., the annular flow channel, can be easily fabricated by an FDM-type printer.

Experiments have demonstrated that the device could flow air out of the annular flow channel in the expected way and that the drying effect was in line with expectations.

Normally, when predicting the drying time, it is necessary to consider not only the constant drying rate but also the critical moisture content and the falling drying rate. However, our approach is in situ hot air flow drying; the drying process is highly transient and hot air tends to be highly concentrated on the material just deposited. Therefore, only the constant drying rate is considered by the following [45]:

(20) $$R_{c}=\frac{\alpha_{c}\left(T_{\mathrm{gas}}-T_{\mathrm{wet}}\right)}{r_{w}}$$

where $\alpha_{c}$ is the combined heat transfer coefficient, $r_{w}$ is the latent heat, $T_{\mathrm{gas}}$ is the dry-bulb temperature, and $T_{\mathrm{wet}}$ is the wet-bulb temperature.

For the extruded thin moist material, the moisture-content and temperature differences are very small and the moisture and temperature distributions are nearly uniform. Furthermore, if given the initial moisture content $\varphi_{w0}$, for the porous ceramics, the rate of decrease in the moisture content $\dot{\varphi_{m}}$ can be linearly estimated by the following [46]:

(21) $$\dot{\varphi_{m}}=0.073R_{c}$$

If we suppose 90% of the moisture content would be dried, the drying time $t_{\mathrm{drying}}$ can be estimated by the following:

(22) $$t_{\mathrm{drying}}=\frac{0.9\varphi_{m0}}{{\dot{\varphi }}_{m}}$$

### 5.3. Synthesis Framework of Full 3D Printability

**Figure 10:**
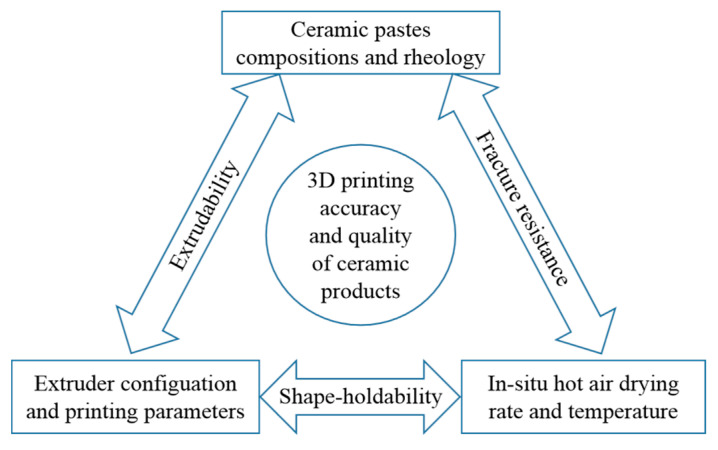
Mathematical equations coupling framework for full three-dimensional printability.

From Equation (1) to Equation (22), we systematically introduce the mathematical representations for the extrusion-based 3D-printing process of ceramic pastes. These mathematical equations can scientifically describe the extrusion dynamics, slurry rheology, drying kinetics, and cracking behavior. In practical engineering, as illustrated in Figure 10, they should be exactly coupled by controllable enabling technologies to ensure high dimensional accuracy and good surface quality.

Looking back at the complexity of 3D printability stated in Section 2.1, apparently, this framework contributes to understanding what is the full 3D printability of the extrusion-based 3D-printing process of ceramic pastes and reduces the blindness of a process system setup. For building a completely programmable and controllable extrusion-based 3D-printing system, the extrudability, shape-holdability and fracture resistance not only need fully mathematical representations but also need wholly enabling technologies. As far as we know, this synthetic framework is the first attempt to provide a fully mathematical survey for the extrusion-based 3D-printing process while most of previous research work mainly focused on one point [47,48,49,50].

### 5.4. Verification of In Situ Hot Air Flow Drying Method

**Figure 11:**
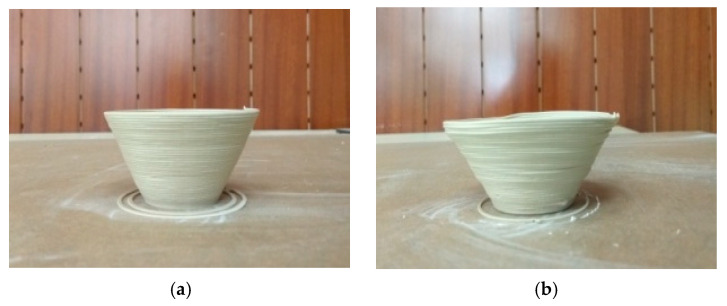
Conical thin-walled benchmark parts with in situ hot air flow drying: (**a**) cone angle as 30° and (**b**) cone angle as 35°.

Experiment 1. First, in order to verify the effect of the developed in situ hot air flow drying method, we used the same conical thin-walled benchmark model and the same process parameters as in the previous experiments referred to Table 1. After using the developed in situ hot air flow drying device, as seen in the Figure 11, the experiments demonstrated that the maximum cone angle of the benchmark part could be up to 35° and the forming process was more stable.

**Figure 12:**
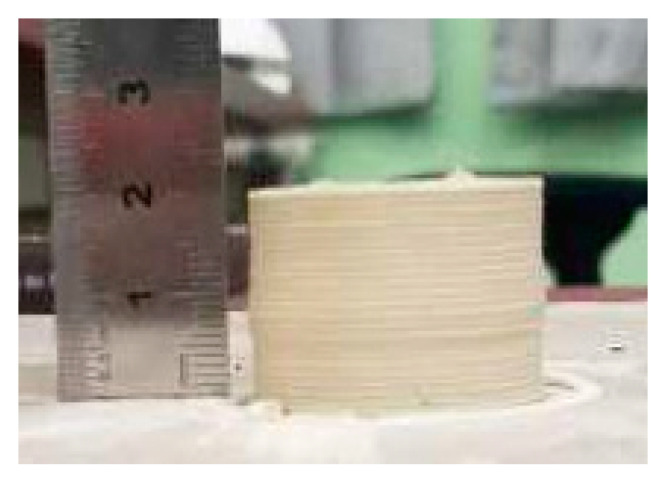
The fully solid cylinder part with in situ hot air flow drying.

Experiment 2. The second verification experiment was to print the fully solid cylinder part, and the same process parameters were set up as in the previous experiment, refer to Figure 5. Due to the assistance of in situ hot air flow drying, the solid cylinder part was well shaped without any deformation, as shown in the Figure 12.

**Figure 13:**
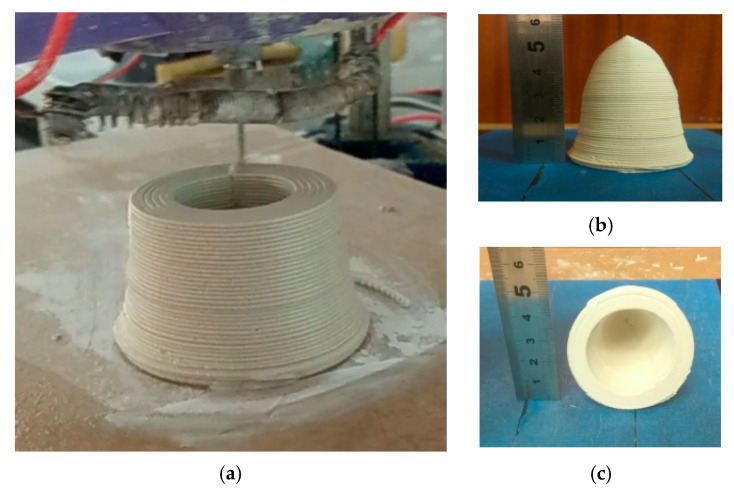
Three-dimensional printing of the fire arrowhead-like object with in situ hot air flow drying: (**a**) extrusion-based 3D printing, (**b**) finalized object, and (**c**) smooth intracavity.

Experiment 3. Furthermore, to fully inspect the shape-holdability of the in situ hot air flow drying method, we also printed a fire arrowhead-like object, as shown in the Figure 13. Clearly, without any support materials, it could be seen that the whole printing process had good long-time shaping stability and that the outer and inner surfaces displayed good quality. This typical object can also be used as an interesting and intuitive case with the hollow ogive cone made by the aqueous-based extrusion process [11] or the similar hollow cone sample made by the freeze-form extrusion fabrication method [14]. This indicated that our given method had the strong capacity to construct objects with more complex structures, e.g., overhanging features.

**Figure 14:**
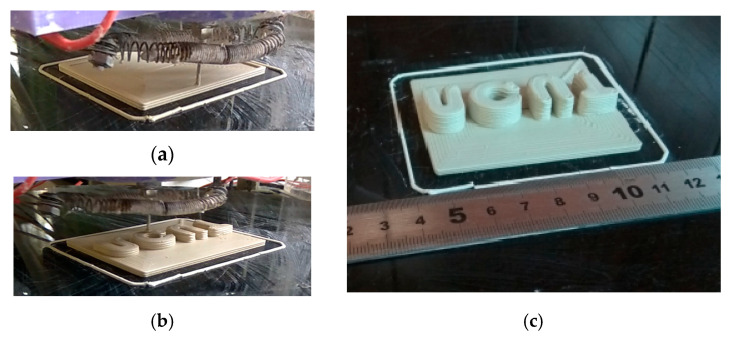
Three-dimensional printing of the multi-parts array with in situ hot air flow drying: (**a**) extrusion-based 3D printing, (**b**) 3D-printing island structures, and (**c**) finalized multi-parts array.

Experiment 4. For a completely programmable and controllable extrusion-based 3D-printing system of ceramic pastes, the dynamically discontinuous extruding ability is also very important, which is necessary for printing one part with many noncontinuous island structures and for the mass customized production of different parts. As shown in Figure 14, one multi-letter array (ncut) part was successfully printed with the online hot air flow drying. Obviously, in this context, the presented in situ hot air flow drying method has an advantage over the uniform layered radiation drying method [30]. This is because the latter is radiation drying in the same layer, which would cause nonuniform moisture gradients of different structural islands.

### 5.5. Shaping Capacity and Accuracy

**Figure 15:**
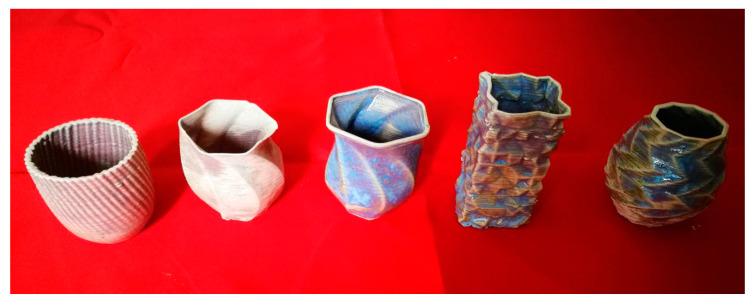
Sintered three-dimensional printed ceramic samples.

Obviously, the in situ hot air flow drying method makes the complex drying kinetics of deposited pastes easily controllable, which can remove about 90 wt.% water content online. The green body only needs a very short time naturally bulk drying of less than three hours before sintering. The above experimental results of thin-walled cone-shape benchmark parts and the fire arrowhead-like object directly demonstrated that the developed method could extend the shaping capacity of the general extrusion-based 3D printing and could reduce crack formation during drying and could increase the surface smoothness. In our self-developed extrusion-based 3D printer, we successfully printed many parts up to the maximum size ∅100 mm×150 mm and the minimum wall thickness 1.2 mm, and some samples are living proofs in Figure 15. The Archimedes density data for our samples showed that these samples averagely achieved about 95% of their theoretical density.

Moreover, we were also very concerned about its shaping accuracy. Through measurements and statistics, the dimensional average error of the solid parts is about 1.5 mm, and the thin-wall parts are about 0.2 mm. For more quantitative research details, refer to our previous publication [39]. The error data also indicated that, during the printing process, the layer-to-layer compaction phenomenon and liquid phase migration phenomenon were difficult to avoid and would cumulatively affect the dimensional accuracy of the deposited portion.

### 5.6. Comparison with Other Shape-Holding Methods

As reviewed in Section 2.2, our field researcher explored many different shape retaining strategies. After above experimental checkouts, it is time to compare our presented methods to other methods. As listed in Table 2, we conducted a comparative analysis among the currently published shape-holding methods and our presented method. Undoubtedly, different shape-holding methods have their own pointcuts, different technical complexity and finite applicability. However, in the comprehensive contexts of environmental pollution, shaping capacity, complexity of implementation, and applicability, it was apparent that our presented method had more comparative advantages over other methods. Certainly, we cannot deny that our method still has a lot of room for improvement for future industrialization of heating manners and drying efficiency.

## 6. Sintering Experiment of 3D-Printed Ceramic Samples

Sintering of ceramic materials is the method involving consolidation of ceramic powder particles by heating the “green” compact part to a high temperature below the melting point, when the material of the separate particles diffuse to the neighboring powder particles. Sintering methods commonly include atmospheric pressure sintering, hot pressing, hot isostatic pressing, and so on. Here, to investigate the sinterability and sintering shrinkage of the extrusion-based 3D-printed green bodies, we used the atmospheric pressure sintering to sinter 3D-printed ceramic samples.

According to our experimental observation, no cracks occurred in all of the 3D-printed ceramic samples. The measurements and statistics showed that there was no dimensional shrinkage of the fully dried 3D-printed ceramic samples after biscuit firing at the temperature 800 °C. Moreover, we also tried to glaze the 3D-printed ceramic vessels as given in the Figure 15. Furthermore, the dimensional shrinkage of the 3D-printed biscuit bodies is about 20% after high-temperature glazed sintering at the temperature 1250 °C [39]. These ceramic products with profiled surfaces were almost impossible to fabricate via traditional craftsmanship. This demonstrates that fully digital and easily available extrusion-based 3D printing of ceramics has great potentials.

## 7. Conclusions

Generally, extrusion-based 3D-printing methods are viewed as one of the most popular approaches to freeform fabrication of ceramic components, but there is still much room for improvement in this type of 3D-printing method, especially in terms of the complexity and dynamics of extrusion behavior. Focusing on explicating what is its full 3D printability and enabling the numerically controlled shape-retention method, this work made new progress based on improvements in the existing extrusion-based 3D-printing research results.

The first contribution is to build the geometrically mathematical model for extrusion-based 3D printing for ceramic paste using infinitesimal method. Nevertheless, this analysis model is steady state with many assumptions, but the companionship among the most important process parameters (i.e., layer height, paste extrusion speed, and scanning speed) is firstly represented by the equilibrium equation of the extrusion process. This formulaic argument contributes to providing theoretical indications for reducing the blindness of experimental optimization. Furthermore, this work systematically provided a fully mathematical framework for the extrusion-based 3D-printing research community [51,52] from several realms: pastes rheology, extrudability, shape-holdability, and drying kinetics. Undoubtedly, this mathematical establishment provides a clearer and more reliable basis for extrusion-based process parameter optimization for ceramic pastes or other similar feedstocks [53].

Further inspired by the generalized drying kinetics of porous materials, a new type of shape-retention method is presented using in situ hot air flow drying. Collectively, it can quickly dry the paste moisture to avoid nonuniform shrinkage stresses and to heighten the critical cracking thickness. Experimental work of the thin-walled cone-shape benchmark parts, the fire arrowhead-like object, and island structures fully demonstrated that the developed method could extend the shaping capacity of general extrusion-based 3D-printing methods, could reduce cracks formation during drying, and could increase the surface smoothness and dimensional accuracy of the 3D-printed parts. In essence, this remodeled method provides an online method of locally uniform extrudate drying in sync with the digital material deposition. The palpably artistic ceramic products with complex profiled surfaces initially highlight the game-changing potentials originating from the fusion of scientific research and technological innovation.

## Figures and Tables

**Table 1:** Experimental conditions and data.

Thin-Walled Benchmark Part	Layer Height	Extrusion Speed		Q_d_ by Equation (1)	μ by Equation (11)	ΔP by Equation (12)	$\dot{\gamma }$ by Equation (13)	τ by Equation (14)	$d_{\mathrm{swell}}$ by Equation (15)	$\tau_{\mathrm{wall}}$ by Equation (16)
		Estimates	Actual							
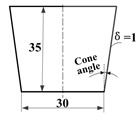	0.8 dmm	2.49 mm/s	2.6 mm/s	2.04 mm^3^/s	144 Pa·s	0.3 MPa	21 1/s	0.0355 MPa	1.16 mm	3.0 × 10^−3^ MPa
	0.7 dmm	2.07 mm/s	2.2 mm/s	1.73 mm^3^/s	142 Pa·s	0.25 MPa	18 1/s	0.0354 MPa	1.12 mm	2.5 × 10^−3^ MPa

**Table 2:** Comparative analysis of different shape-holding methods.

Shape-Holding Methods	Principle Effects	Main Advantages	Implicit Disadvantages
Without auxiliary shape holding method [11,22]	–	Extrusion-based layered forming process with directly atmospheric drying is an eco-friendly process to create 3D objects.	This process has large uncontrollable variability owing to paste property variability. The green body needs a long-time for bulk drying before sintering.
Deposition in freezing environment [14,26]	Physical	The freezing environment using a liquid nitrogen injection system enabled the aqueous pastes to solidify at temperatures below the freezing point of water, thus avoiding part deformation and enabling fabrication of larger parts.	Besides the freezing system, a heating jacket was also needed to keep paste temperatures above the freezing point of water until it was deposited. Before sintering, the moisture still needs a long drying time.
Using removable support structures [34]	Physical and Chemical	To make complex ceramic components, double-head extrusion was used to print two materials: one to form the main part and another to form the support structure.	The support material must meet the typical requirements of 3D printability. The support material should be compatible with the main material and easily removed in postprocessing.
Using chemical binders [13,15]	Chemical	Concentrated colloidal gels with tailored viscoelastic properties were designed to form self-supporting features and were deposited in a layer-by-layer sequence to directly write the desired 3D objects.	The gelling of ceramic colloids or polymers must exhibit a controlled viscoelastic response and contain high colloid volume fractions to minimize shrinkage. During sintering, the gels may release harmful chemicals.
Using thermally induced gelation [30]	Chemical	Shaping of ceramics by thermally induced gelation of methyl cellulose is eco-friendly as it is a naturally occurring polymer and is effective in very low concentrations.	The part while printing also needs to be exposed to the gelation temperatures using a honeycomb-based energy efficient air heater. Before sintering, the moisture still needs bulk drying.
Uniform layered radiation drying [29]	Physical	Optimal partial drying with the infrared lamp enables strong bonding between layers. The liquid oil surrounding the part could preclude crack formation, warpage, and moisture gradient in the part.	The oil filling and infrared heating systems make the whole system too complicated. In postprocessing, the oil bath needs draining and the remaining water further needs a long drying time.
In situ hot air flow drying	Physical	The in situ hot air flow drying device provides online local drying on the deposited spot in sync with the digital material deposition. The auxiliary heating system is very simple and eco-friendly. The green body needs a very short bulk drying time before sintering.	According to current experiments, no obvious disadvantages were found. In the future, we will explore more precise regulations of heating manners and drying efficiency.

## Data Availability

All data generated or analyzed during this study are included in this article.

## Nomenclature

ABEF	Aqueous-Based Extrusion Fabrication
AM	Additive Manufacturing
CODE	Ceramic On-Demand Extrusion
DED	Direct Energy Deposition
DIW	Direct Ink Writing
FDC	Fused Deposition of Ceramics
FDM	Fused Deposition Modeling
FEF	Freeze-Form Extrusion Fabrication
ME	Material Extrusion
RC	Robocasting
SLM	Selective Laser Melting
LPM	Liquid Phase Migration
